# Chemotherapy in vivo against M109 murine lung carcinoma with cytochalasin B by localized, systemic, and liposomal administration

**DOI:** 10.1007/s10637-014-0202-6

**Published:** 2015-01-06

**Authors:** Matthew Trendowski, Joan M. Mitchell, Christine M. Corsette, Christopher Acquafondata, Thomas P. Fondy

**Affiliations:** Department of Biology, Syracuse University, 107 College Place, Syracuse, NY 13244 USA

**Keywords:** Cytochalasin B, Liposome encapsulation, Lung carcinoma, Metastasis

## Abstract

Cytochalasin B is a potentially novel microfilament-directed chemotherapeutic agent that prevents actin polymerization, thereby inhibiting cytokinesis. Although cytochalasin B has been extensively studied in vitro, only limited data are available to assess its in vivo potential. Cytochalasin B was administered to Balb/c mice challenged i.d. with M109 murine lung carcinoma to determine whether the agent could affect an established i.d. tumor when the compound is administered s.c. in the region of the i.d. tumor, but not in direct contact with it. Cytochalasin B was also administered either i.p. or s.c. at a distant site or i.v. to determine whether it could affect the long-term development of an established i.d. tumor. Cytochalasin B was then liposome encapsulated to determine whether the maximum tolerated dose (MTD) of the compound could be increased, while reducing immunosuppression that we have previously characterized. Liposomal cytochalasin B was also administered to mice challenged i.d. with M109 lung carcinoma to assess its chemotherapeutic efficacy. The results can be summarized as follows: 1) cytochalasin B substantially delayed the growth of i.d. M109 tumor nodules, inhibited metastatic progression in surrounding tissues, and produced long-term cures in treated mice; 2) liposomal cytochalasin B increased the i.p. MTD by more than 3-fold, produced a different distribution in tissue concentrations, and displayed antitumor effects against M109 lung carcinoma similar to non-encapsulated cytochalasin B. These data show that cytochalasin B exploits unique chemotherapeutic mechanisms and is an effective antineoplastic agent in vivo in pre-clinical models, either in bolus form or after liposome encapsulation.

## Introduction

Cytochalasins are mycogenic toxins known to disrupt the formation of actin polymers, thereby preventing the formation of functional microfilaments. These congeners are characterized by a highly substituted perhydro-isoindolone structure that is typically attached to a macrocyclic ring [1]. More than 60 different cytochalasins from several species of fungi have been classified into various subgroups based on the size of the macrocyclic ring and the substituent of the perhydroisoindolyl-1-one residue at the C-3 position [2]. However, the most studied congener in regards to antineoplastic activity has been cytochalasin B. As a microfilament-disrupting agent, cytochalasin B alters cell motility, adherence, secretion, drug efflux, deformability, morphology, and size, among many other effects [1, 2]. The effects of cytochalasin B on cell adherence include alteration of cell junctions, leading to changes in tissue physiology and transport.

Perhaps the best characterized cellular property of cytochalasin B is its ability to prevent formation of the contractile ring, as well as development of the cleavage furrow, thereby inhibiting cytokinesis [3, 4]. While normal proliferating cells enter the G_0_ resting state until sufficient actin levels have been gathered for successful cytokinesis, malignant cells typically continue to progress through the cell cycle, resulting in the formation of enlarged, multinucleated cells. The multinucleation phenomenon is almost exclusively observed in neoplastic cells, potentially representing a neoplastic-targeting feature of cytochalasin B, as well as other congeners [5–12]. This apparent partial specificity of cytochalasin action on neoplastic cells is consistent with a separate body of evidence showing substantial differences in microfilament biochemistry between neoplastic and comparable non-neoplastic cells [13]. These differences in microfilament structures may be related to key neoplastic characteristics, including altered adherence, anchorage independent growth, invasiveness, and altered plasma membrane cytoskeletal interactions involving expression of oncoproteins [14].

These observations lead us to propose that cytochalasin B might affect experimental cancer chemotherapy model systems in vivo, either as single agents, or as agents amplifying responses of known chemotherapeutic agents. Mechanisms of action of microtubule-directed or of nucleic acid-directed agents might be affected by microfilament dependent phenomena (see Ref 4 for detailed explanations of potential drug synergy). We propose that the cytochalasins as microfilament-directed agents may enhance the antineoplastic activity of other cytoskeletal-directed agents such as the microtubule-disrupting vinca alkaloids, or the microtubule-stabilizing taxanes.

In previous work, we developed procedures for the preparation of cytochalasins in quantities sufficient to support comprehensive in vivo evaluation, and we have defined the conditions for the formulation, administration, and limiting doses for intraperitoneal (i.p), intravenous (i.v)., and subcutaneous (s.c.) treatment [15, 16], as well as the kinetics of tissue distribution to 14 tissues after i.p. treatment [15]. We have established that cytochalasin B administered s.c. in mice peritumorally to s.c. challenges of B16F10 murine melanoma, or of M109 murine lung carcinoma is able to inhibit invasion and spontaneous lung metastases, and in the case of both models to increase life span [16]. Further, our in vitro and in vivo analyses of B16F10 cells suggest that the antitumor effects of localized cytochalasin B do not arise from gross toxicitiy, but rather may depend on other features of host-tumor interaction.

However, the immunosuppressive effects of cytochalasin B in vivo raise concern. While cytochalasin B has demonstrated substantial antitumor activity, the maximum tolerated dose (MTD) is limited due to immunosuppression observed in the spleen, coupled with marked splenomegaly [15, 16]. We have demonstrated that this immunosuppression can be reversed through the introduction of human recombinant interleukin-2 (rhIL-2) [17], but developing a safer vehicle by which to transport cytochalasin B to malignant cells is pivotal for its development as a chemotherapeutic agent. Liposomes have been extensively examined for their abilities to improve drug delivery, and have been used for a substantial variety of medicines, including cytotoxic agents, antibiotics and antifungal agents [18]. In regards to cancer therapy, liposome encapsulation has been shown to reduce side effects of a variety of chemotherapeutic agents, as well as promote targeted tumor damage due in part to their ability to substantially aggregate at tumor sites by leaking through pores and defects in tumor capillary endothelium [18]. In fact, cytochalasin D, a congener of cytochalasin B that has a toxicity in mice that is 20-fold higher than does cytochalasin B [19], has demonstrated reduced toxicity, as well as increased efficacy after being encapsulated in polyethylene glycol (PEG) coated liposomes [20]. It may be possible to dramatically increase the MTD of cytochalasin B, while reducing its immunosuppressive effects through liposome encapsulation.

Therefore, this study seeks to determine the following: 1) whether cytochalasin B in vivo can affect an established intradermal (i.d.) tumor when the compound is administered s.c. in the region of the i.d. tumor, but not in direct contact with it; 2) whether cytochalasin B administered either i.p. or s.c. at a distant site can affect the long-term development of an established i.d. tumor; 3) whether cytochalasin B systemically administered i.v. can affect the subsequent development of a localized, potentially invasive and spontaneously metastatic i.d. tumor; 4) whether the MTD of cytochalasin B in mice can be elevated through liposome encapsulation; and 5) whether the tissue distribution and anticancer activity of liposomal cytochalasin B is similar to that of the non-encapsulated compound.

## Materials and Methods

### Conversion of M109 to Continuous Culture (M109c)

M109 murine lung carcinoma cells were converted to continuous culture using methods previously described [21]. As in the previous paper of this series [21], comparison between animals challenged i.d. with M109c cells and matched groups challenged with in vivo passaged M109 cells showed that the challenges were indistinguishable in terms of growth kinetics, invasion, metastasis, and host survival.

### M109 Lung Carcinoma In Vivo

M109 murine lung carcinoma was maintained as a spontaneously invasive and metastatic model by serial passage s.c. in Balb/c mice as previously detailed [4, 22], except that the passage challenge was 2 × 10^5^ trypan blue negative cells. Prolonged tumor passage i.d. afforded a gradually less invasive and metastatic variant that permitted longer term measurements of a completely localized primary i.d. nodule. When this subline was used, the tumor challenge is designated M109ni.

For chemotherapy testing, Balb/c mice under isoflurane anesthesia (Sigma-Aldrich Corp., St. Louis, MO, USA) were challenged i.d. on the lower right ventral quadrant over the peritoneum after first removing hair with a chemical depilatory agent. The challenge dose was 2 × 10^5^ trypan blue negative cells in a volume of 200 μl. Tumor cells were obtained from early s.c. tumor in vivo passaged donors (M109), from prolonged i.d. passaged donors (M109ni), or from continuous cultures of M109 lung carcinoma cells (M109c). Tumor growth was monitored by daily caliper measurements, which were facilitated by the localized growth of the i.d. challenge contained entirely in a region initially free of hair. Whereas invasion took place with the M109 or M109c nodules, localized tumor growth tended to become variable after about 14 days, when invasion accompanied by systemic metastatic spread had occurred. Non-invasive and non-metastatic M109ni cells permitted the monitoring of primary tumor growth until reaching a limiting predetermined tumor size (18 mm in any one direction) or until host conditions dictated termination. Therefore, survival in the M109 and M109c challenged groups is based on systemic tumor effects, whereas survival in the M109ni challenged groups is an extension of primary tumor measurements. Effects on primary tumor appearance, growth rate, invasion, and metastases to the lungs, liver, kidneys, pancreas and spleen were determined as previously detailed for s.c. challenges with M109 tumor [16].

### Cytochalasin B Preparation, Bolus Formation and Administration

Cytochalasin B was prepared from mold mattes of *Drechslera dematioidea* (ATCC 24346) as previously described, and purified by preparative thin layer chromatography to greater than 99 % homogeneity after recrystallization from chloroform. The compound was prepared in suspension form in 2 % carboxymethyl cellulose 1 % tween 20 for s.c. administration, and in solution in 33 % ethanol: 0.9 % NaCl solution for i.v. use as previously described [15, 16].

### Liposomal Cytochalasin B Preparation

Liposomal cytochalasin B was prepared by suspending 2.0 mg cytochalasin B in 2.0 ml chloroform containing 200 mg of egg phosphatidylcholine (Sigma-Aldrich Corp.). The solution was pipetted into a round bottom flask and dried to a thin film. The film was then resuspended in 5 ml phosphate buffered saline (Sigma-Aldrich Corp). The resulting suspension was left to sit for 1 h before being extruded five times through a 5.0 μm polycarbonate filter in accordance to LUVET (large unilamellar vesicles made by an extrusion technique), forming large, unilamellar vesicles. The above mentioned protocol was then repeated using 200 mg total of a 70:30 mixture of egg phosphatidylcholine: dimyristoylphosphatidylglycerol.

### Determining the Extent of Reduction in Immunosuppression Potentiated by Liposomal Cytochalasin B

To determine whether liposomal cytochalasin B had less of an immunosuppressive effect than its natural counterpart, dose–response and time-course study of the influence that i.p. bolus injection had on spleen response to an allogeneic sensitizer and target tumor cell in vitro were performed as previously described [17]. Splenic lymphocytes from mice were removed following natural or liposomal cytochalasin B treatment and exposed to X-radiated P815 murine mastocytoma tumor antigen as described in [23] with the modifications presented in [24]. As in [17], the cytotoxic activity of sensitized spleen cells was determined in a standard 4 h Cr-release assay. Spleen cell number after sensitization and washing was checked by triplicate hemacytometer counts to confirm the final effector: target (E:T) ratios to permit comparisons among the treatment groups at equivalent ratios. The percentage of ^51^Cr release was determined through the formula ^51^Cr release = [(^51^Cr supernatant/(^51^Cr pellet + ^51^Cr supernatant)] × 100. The percentage of specific Cr release represents values obtained with immune effectors minus values obtained from control lymphocytes cultured for 4 days in the absence of P815 antigen. Control lymphocytes were prepared from each of the groups treated at various cytochalasin B or liposomal cytochalasin B concentrations, or with the vehicle.

### Statistics

Survival analysis used the Cox-Mantel test as detailed by Lee [25]. Effects on tumor growth rates were evaluated by Repeated Measures Analysis of Variance using SAS software. Test of hypotheses for between subject effects were applied, and of time vs. group interactions using the Geisser-Greenhouse adjustment in cases where tests of orthogonal components showed absence of sphericity. Differences in lung metastases between treated and control groups were evaluated with a Student-Fisher *t*-test.

## Results

### Conversion of In Vivo Passaged M109 Cells to Continuous Culture (M109c) Cells

M109c cells seeded at 4 × 10^4^ cells/cm^2^ grew as attached cells and reached confluency at 2 × 10^5^ cells/cm^2^ after 5 days. The attached cells were compared to the in vivo passaged cells for tumorigenicity defined as fraction of animals developing tumor and time to appearance of palpable tumor nodules, growth rate, invasion, and formation of spontaneous metastases. Growth curves in vivo for i.d. tumors produced by challenge with 2 × 10^5^ M109c cells were fully superimposable for 19 days with growth curves obtained in mice challenged with 4 × 10^5^ M109c trypan blue negative in vivo passaged M109 cells. After 21 days, spontaneous lung metastases had a mean value of 80.3 nodules per mouse (SEM = 19.7) for tumors produced by the attached cells, compared to a mean of 86.6 nodules per mouse (SEM = 13.6) for tumors produced from in vivo passaged cells. Neither of the tumor sources produced significant numbers of metastases to the liver, kidney, spleen, or pancreas. Tumors grew and invaded in all animals challenged i.d. with either tumor source.

### Effects of S.C. Cytochalasin B Bolus Suspension on Established I.D. M109 and M109c Tumors

**Fig. 1:**
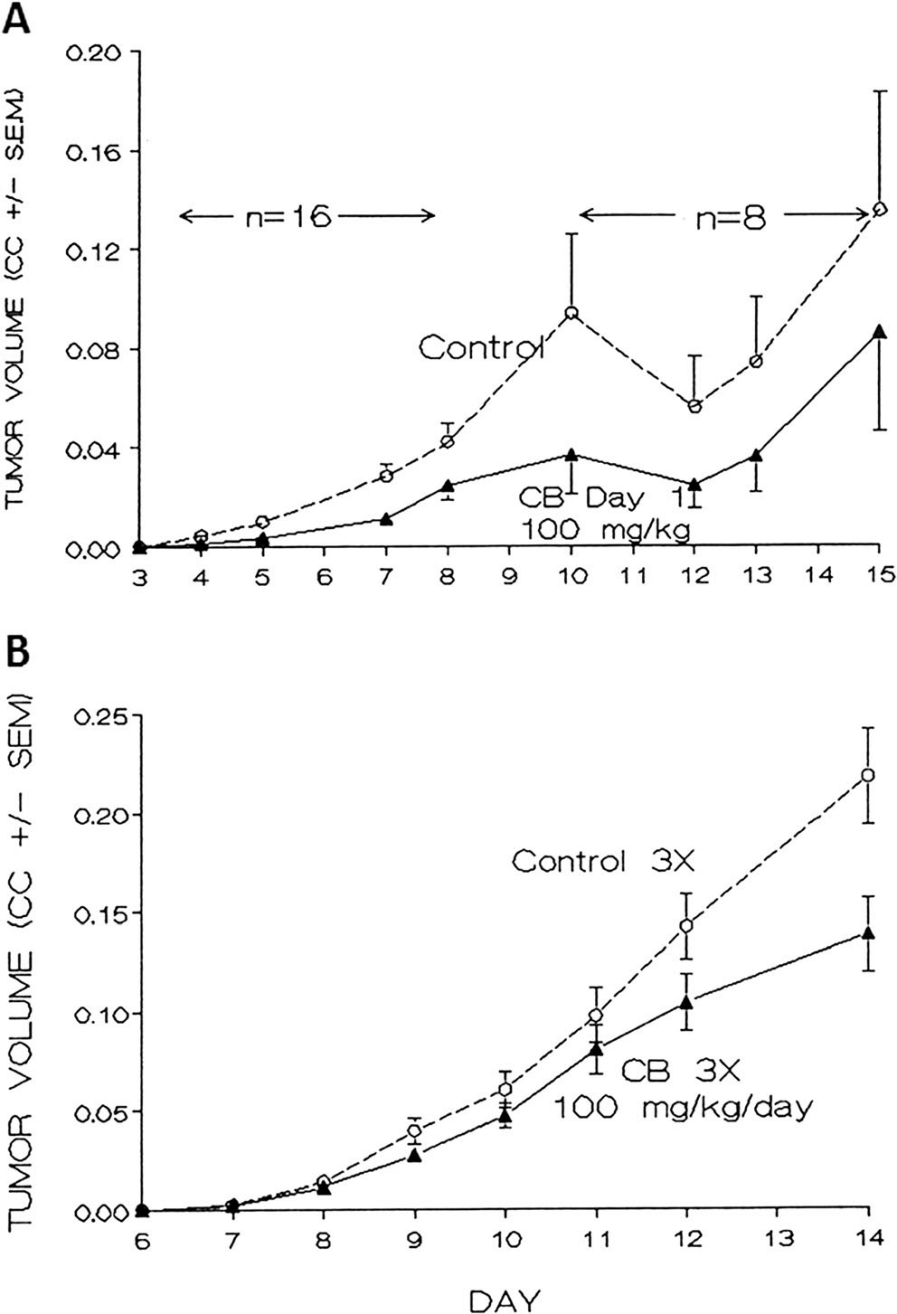
Mean volume of M109 lung carcinoma intradermal nodules treated with subcutaneously administered cytochalasin B. Balb/c mice were administered s.c. cytochalasin B (CB) 100 mg/kg/day in 2 % CM-cellulose/1 % Tween 20 after challenge with 2 × 10^5^ M109 cells on day 0. Control indicates mice treated only with the vehicle in which CB was administered. CC = cm^3^. **a** Single dose treatment with CB day 1. There were 16 mice per group, with half being sacrificed on day 8, the remainder being sacrificed on day 16; *p* < 0.01 for between subject effects, and *p* < 0.08 for time vs. group interaction. **b** Multiple dose treatment of established tumor nodule with CB days 7, 9, and 11. There were 10 mice per group; *p* < 0.05 for between subject effects, and *p* < 0.01 for time vs. group interaction. *Bars* reflect standard error of the mean (SEM) of the treatment groups

Cytochalasin B administered s.c. as a single bolus dose at 100 mg/kg (Fig. 1a), or three such doses spaced at two-day intervals (Fig. 1b) peritumorally to nascent day 1 challenges (Fig. 1a) or to established i.d. (0.5 mm diameter) nodules (Fig. 1b) of M109 tumor, significantly inhibited the rate of tumor growth in treated animals. Multiple dose treatments produced growth inhibitory effects statistically more significant than single dose treatments. Multiple dose treatment increased median survival by 33 % in treated animals (15 days in controls, compared to 20 days in the treated group, significant at the 0.05 level by *U*-test. The mean number of metastases to the lungs at the respective terminal stages was not significantly affected.

Treatment of established i.d. tumor nodules of M109c on days 6, 8, and 10 after tumor implantation with bolus cytochalasin B under conditions identical to those used in Fig. 1b produced significant inhibition of tumor growth rate (*P* < 0.001 between subject effects; p, 0.01 time vs. group interaction by repeated measures analysis of variance) (data not shown). Spontaneous lung metastases determined on day 17 showed a mean number of lung nodules in controls of 23.5 nodules per mouse (SEM = 9.7) compared to 8.3 nodules per mouse (SEM = 4.1) in the treated group (*n* = 10). This inhibition of spontaneous lung metastases formation was significant at the *P* < 0.05 level.

### Effects of I.V. (Systemic) Cytochalasin B on I.D. Challenge with M109c Tumor Cells

**Fig. 2:**
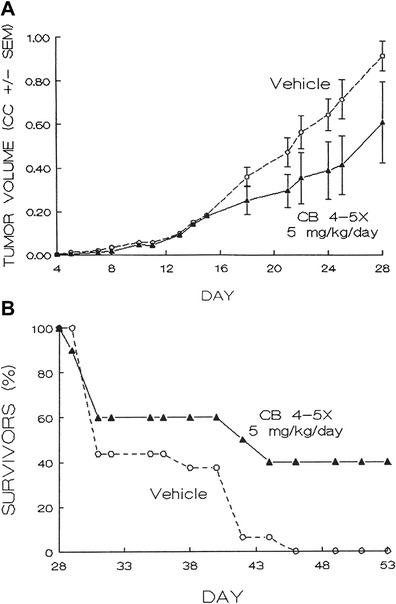
Intravenously administered cytochalasin B against M109 murine lung carcinoma. **a** Effect of intravenously administered cytochalasin B on growth of intradermal M109 carcinoma nodules. 10 mice received at least 4 injections of CB at 5 mg/kg in 33 % ethanol:0.9 % NaCl solution i.v. on days 1–5 following challenge on day 0 with 2 × 10^5^ M109c cells i.d. 16 Balb/c mice received vehicle i.v. as a control. CC = cm^3^. The CB-treated group was significantly different from the controls; *p* < 0.02 for between subject effects, *p* < 0.05 for time vs. group interactions. *Bars* reflect SEM of the treatment groups. **b** Effects of intravenously administered cytochalasin B on survival of Balb/c mice. Both treated and control mice were administered the same concentrations of cytochalasin B or vehicle-only as shown in Fig. 2a. Survival of treated mice (*n* = 10) was significantly different from controls (*n* = 16); *p* < 0.05, as assessed by a Cox-Mantel test

Animals challenged with M109c cells i.d. (day 0) were treated daily with i.v. cytochalasin B solution in ethanol-saline at 5 mg/kg/day for 4 or 5 successive days beginning 1 day after tumor implantation. Figure 2a shows a strong systemic effect on tumor growth rate produced by i.v. cytochalasin B. It is apparent from Fig. 2a that the growth inhibition is not significant until after day 15, 10 days after the final i.v. drug treatment. The potential implications of this observation will be addressed in the discussion.

As detailed in Fig. 2a, animals treated with i.v. cytochalasin B showed a marked increase in survival duration, and 4 out of 10 animals were tumor-free on day 53 (Fig. 2b). This effect is significant at the 0.03 level by a Cox-Mantel test. Lung metastases at the terminal stages measured in vehicle controls showed a mean value of 73.7 nodules per mouse (SEM = 9.4) compared to a value of 22.7 nodules per mouse (SEM = 3.7), a 70 % reduction, for the six treated animals that succumbed to the tumor challenge (*P* < 0.05). When all 10 treated animals are used in the determination of “terminal” lung metastases, including the 4 long-term tumor-free survivors, the mean becomes 13.6 nodules per mouse (SEM = 4.3) an 82 % reduction compared to the vehicle controls.

### Effects of I.P. and of Distal S.C. (Non-Peritumoral) Cytochalasin B on Established I.D. Tumor Nodules

**Fig. 3:**
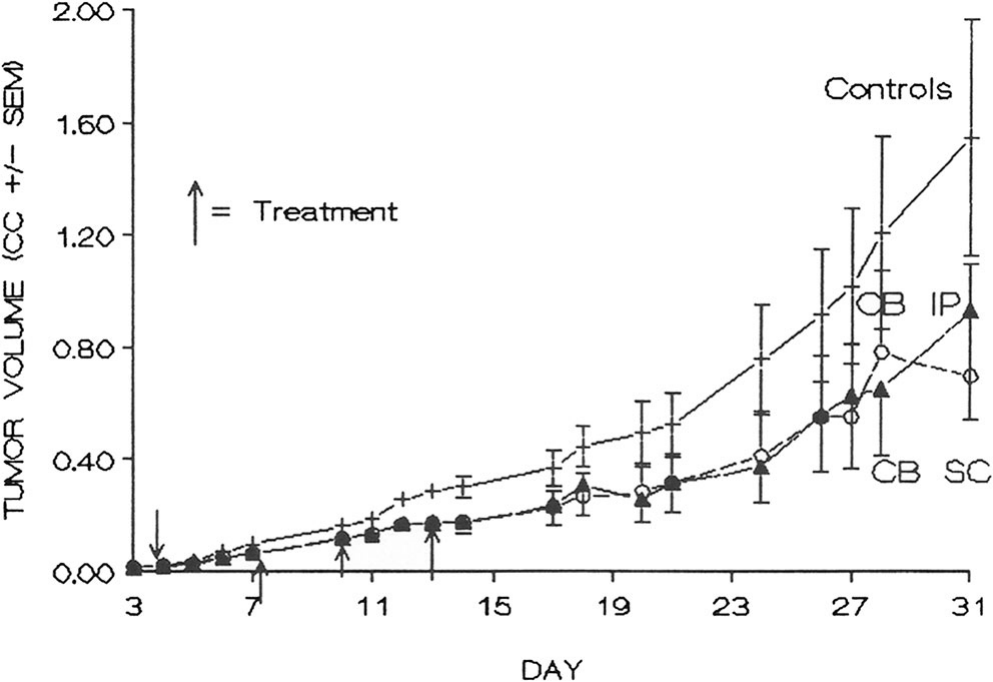
Effects of cytochalasin B administered intraperitoneally or non-peritumoral subcutaneously on long-term growth of established M109ni tumor nodules. Cytochalasin B was administered at 50 mg/kg/day for i.p. treatments, and 100 mg/kg/day for s.c. treatments on days 4, 7, 10 and 13. CC = cm^3^. There were 10 mice per treatment group; i.p. treated group *p* < 0.07, s.c. treated group *p* < 0.09. Combined i.p. and s.c. treated groups *p* < 0.04 for between subject effects

Mice were challenged with M109ni cells i.d., and then stratified into two treatment and one control groups, 10 animals per group, according to tumor size on day 3. Treatments were given on days 4, 7, 10, and 13; 50 mg/kg/day i.p., or 100 mg/kg/day s.c., with cytochalasin B in carboxymethylcellulose-tween suspension as detailed in the materials and methods section. The i.d. tumor challenge was in the lower left anterior quadrant over the peritoneal cavity. Cytochalasin B treated i.p. or s.c. was monitored to day 35. Figure 3 shows the results obtained. Marked systemic activity is apparent as tumor growth inhibition with both i.p. and non-peritumoral s.c. treatment. Statistical analysis of the separate i.p or s.c. treated groups compared to controls to day 24 showed marginal significance (between subject effects *p* < 0.07 and *p* < 0.09 respectively). Combined i.p. and s.c. treated groups compared to controls to day 24 was statistically significant (between subject effects *p* < 0.05).

### Efficacy of Encapsulating Cytochalasin B in Liposomes

**Fig. 4:**
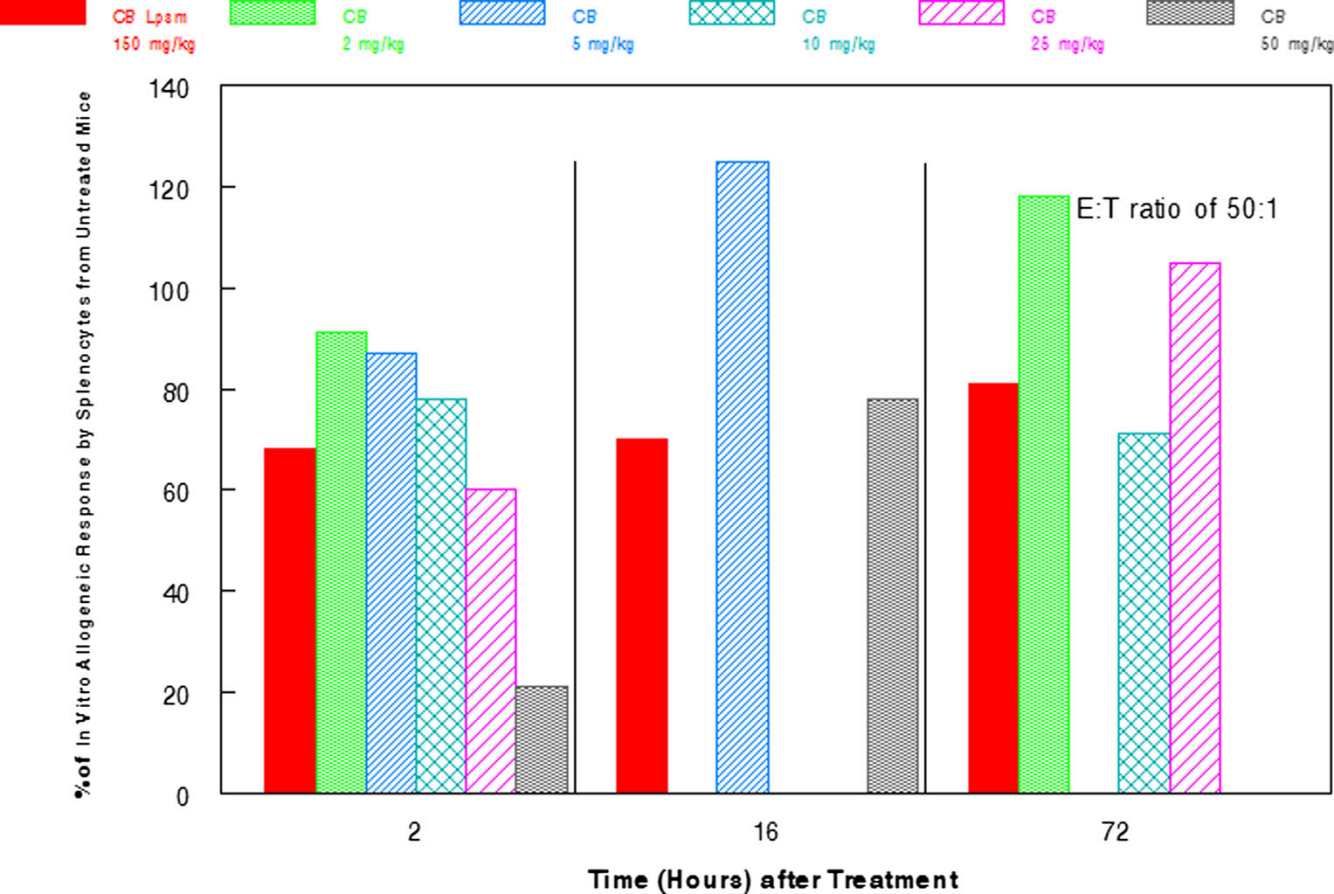
Time course of effect of cytochalasin B/liposomal cytochalasin B on allogeneic response in vitro for E:T of 50:1. Cytochalasin B (CB) or liposomal cytochalasin B (CB Lpsm) was injected i.p. into mice. Spleen lymphocytes were then recovered and sensitized to X-radiated P815 murine tumor antigen for 4 days. The cytotoxic activity of sensitized spleen lymphocytes was determined in a standard 4 h Cr-release assay, and then compared to control lymphocytes from untreated mice or those treated with the vehicle, which was shown not to have an immunosuppressive effect

**Fig. 5:**
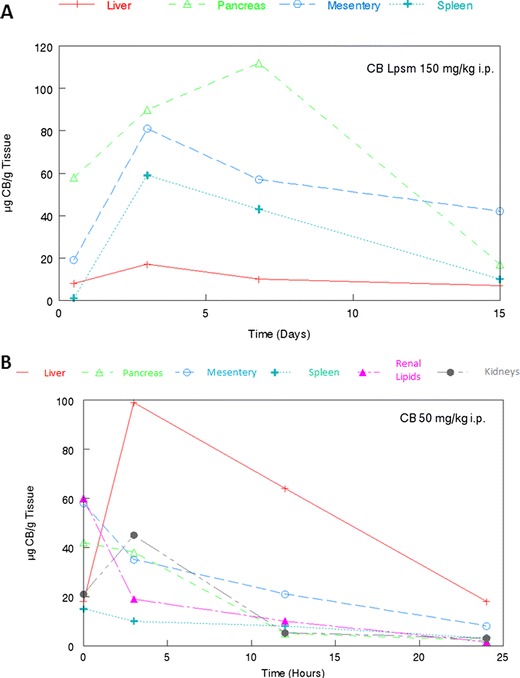
Comparison of tissue distribution for liposomal and natural cytochalasin B. **a** 150 mg/kg liposomal cytochalasin B (CB Lpsm) i.p. in Balb/c mice. **b** 50 mg/kg Natural cytochalasin B (CB) i.p. in Balb/c mice. Analyses were performed by analytical high performance liquid chromatography (HPLC)

As previously shown [17] 50 mg/kg cytochalasin B administered i.p. in mice produced a marked inhibition in the allogenic response to a P815 antigen. By contrast, liposomal cytochalasin B was well tolerated in mice at 150 mg/kg i.p (Fig. 4). Although immunosuppression was noticeable with 150 mg/kg cytochalasin B, it was not as dramatic as the natural compound at only 50 mg/kg. In addition, liposomal cytochalasin B demonstrated marked differences in tissue distribution, as assessed by Balb/c mice (Fig. 5a) in comparison with tissue distribution of non-encapsulated cytochalasin B (Fig. 5b). Liposomal cytochalasin B sustained a high concentration in the mesentery and pancreas, while non-encapsulated cytochalasin B localized in the liver. The time of distribution for liposomal cytochalasin B was also markedly different from non-encapsulated cytochalasin B. Liposomal cytochalasin B was measurable for more than 2 weeks in mesentery, pancreas, and spleen, whereas non-encapsulated cytochalasin B was cleared from four out of six tissues examined within 2 days. Non-encapsulated cytochalasin B was measurable at only very low levels in the liver and mesentery after 2 days.

**Fig. 6:**
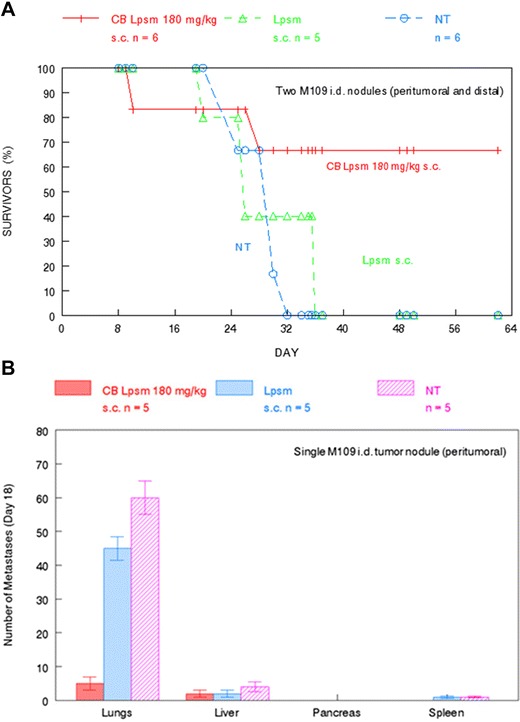
In vivo activity of liposomal cytochalasin B against M109 lung carcinoma. **a** Longitudinal analysis of tumor-bearing mice (peritumoral and distal) survival after treatment with 180 mg/kg CB Lpsm s.c. 8 days after tumor challenge. Size of each treatment group is indicated in the figure. **b** A separate cohort of tumor-bearing mice (peritumoral) were treated with 180 mg/kg CB Lpsm s.c. 8 days after tumor challenge to determine whether CB Lpsm could reduce the number of metastases found in vehicle-only treated and untreated mice 18 days after tumor challenge. *NT* not treated. Size of all treatment groups are indicated in the figure. *Bars* reflect SEM of treatment group

**Fig. 7:**
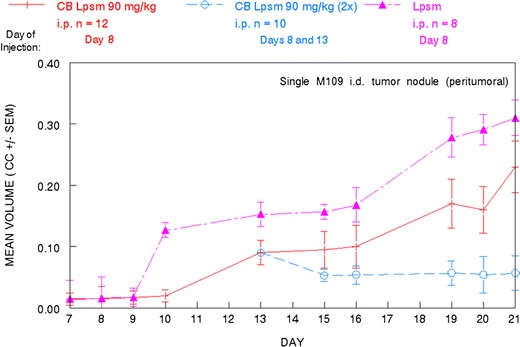
Delay in M109 lung carcinoma tumor growth following different liposomal cytochalasin B treatment protocols. 90 mg/kg CB Lpsm was administered i.p. to tumor-bearing mice (peritumoral) 8 days after the initial tumor challenge. In another cohort, an additional 90 mg/kg CB Lpsm was administered i.p. 13 days after tumor challenge. CC = cm^3^. Size of all treatment groups are indicated in the figure. *Bars* reflect SEM of each treatment group

One hundred eighty milligram/kilogram liposomal cytochalasin B s.c. had considerable in vivo anticancer activity against M109 lung carcinoma. There was a 66.7 % survival rate at least up to day 62 for mice challenged with two i.d. tumor nodules in the cohort treated with liposomal cytochalasin B on day 8 (Fig. 6a). This is a highly significant prolonging of life expectancy, as all controls were dead by day 36. In addition, the same dose of liposomal cytochalasin B markedly reduced the extent of lung metastases observed in Balb/c mice 18 days after challenge of a single i.d. tumor nodule (Fig. 6b). It is unclear whether this ability would hold true in other tissues, as the challenge did not produce significant metastases in the liver, pancreas, or spleen. A particularly intriguing finding was that 90 mg/kg liposomal cytochalasin B administered twice i.p. on days 8 and 13 to tumor-bearing mice with a single i.d. nodule was able to preclude further tumor growth and produce actual tumor shrinkage after the second injection (Fig. 7). A single treatment with liposomal cytochalasin B at 90 mg/kg on day 8 inhibited tumor growth compared to empty liposomal controls, but the tumor continued to grow.

## Discussion

The novel and pleotypic effects of cytochalasins on cells and tissues in vitro have prompted us to predict that these agents will exhibit measurable antitumor activity in vivo by mechanisms which do not depend extensively on gross toxicity [16]. Further, the peculiar properties of cytochalasins suggest that they may be as effective as, or even more effective against solid tumor masses than against free cell neoplasms, because the cell and tissue features affected by cytochalasins are likely to be more important in organized tumor tissue. The relatively low host toxicity exhibited by cytochalasins B is favorable for obtaining significant in vivo preclinical activity against neoplastic growths. Antineoplastic activity is supported by this study, by our previous studies [16, 21], and in the early experience of others in the use of cytochalasins in vivo [26, 27].

Cytochalasin B administered peritumorally in the same compartment [16], locally in separate compartments, or systemically i.p., s.c., or i.v. is active against established tumor nodules, whereas it is inactive when administered i.p. against early i.p. challenges with ascites cancers [16]. This suggests there are some effects on developing solid tumors that may not be as important or as effective at earlier free-cell stages immediately after tumor suspension implantation. It is also evident from Fig. 2a, that i.d. tumor growth inhibition produced by i.v. administration of cytochalasin B does not become significant until after day 15, 10 days after the final i.v. treatment. This delayed effect appears long after drug clearance, and suggests an indirect mechanism relating to host response, or to tissue architecture in developing tumor nodules that may not necessarily rely on immediate acute cytotoxicity.

In our previous study [16], we observed that cytochalasin B administered i.p. was ineffective as a bolus drug suspension in mice challenged i.p. with suspensions of M109 lung carcinoma, or of B16F10 melanoma. By contrast, s.c. challenges of either tumor model exhibited marked sensitivity to peritumoral treatment with s.c. cytochalasin B. In the case of s.c. B16F10 tumor treated with s.c. cytochalasin B, analysis of tumor cell viability, clonogenicity, and tumorigenicity, after drug treatment in vivo demonstrated no evidence of measurable cytotoxicity in tumors whose growth was markedly inhibited [16]. These observations are consistent with a mechanism of antitumor activity for cytochalasin B that is not dependent on gross toxicity.

The s.c. peritumoral activity of cytochalasin B against s.c. nodules of M109 lung carcinoma has now been extended to established i.d. nodules of M109 tumor where the agent is administered s.c. peritumorally, and to models where direct contact between s.c. cytochalasin B and the i.d. tumor nodule does not occur (Fig. 1a and b). Cytochalasin B administered in a multiple dose protocol either s.c. at a site distant from an established i.d. tumor nodule, or i.p. against an established i.d. tumor is also able to substantially inhibit tumor growth (Fig. 3). These results indicate that direct drug-tumor contact is not needed in order for cytochalasin B to achieve a chemotherapeutic response, thereby demonstrating systemic antitumor activity.

Systemic antitumor activity elicited by cytochalasin B against nascent M109 i.d. tumor nodules is confirmed in Fig. 2 which show that cytochalasin B administered i.v. to mice challenged i.d. with M109 tumor cells inhibits tumor growth rate and formation of spontaneous lung metastases, as well as extends host survival with a 40 % (4/10) cure rate. This again demonstrates that direct contact between the administered drug and tumor challenge is not required to produce antitumor activity. The fact that low levels of cytochalasin B administered i.v. (20–25 mg/kg over a 4–5 day period) is active against M109 lung carcinoma, and that the compound does not exhibit activity until 10 days after final i.v. treatment (Fig. 2a), argues against a direct cytotoxic effect. This further supports the postulation that cytochalasin B has an indirect effect on tumor growth, or affects host response that inhibits tumor growth by a delayed mechanism.

Although the anticancer activity of cytochalasin B is substantial, it may be beneficial to encapsulate the agent in liposomes before it is administered in vivo. A three-fold increase in the i.p. MTD compared with non-encapsulated cytochalasin B was achieved by the liposomal derivative, with less immunosuppression being observed at this elevated dose (Fig. 4). In addition, liposomal cytochalasin B appears to have a much longer time of tissue distribution, as well as a different tissue distribution compared to non-encapsulated cytochalasin B (Fig. 5). Liposomal cytochalasin B also demonstrated marked anticancer activity against M109 lung carcinoma, as assessed by a prolonging in life expectancy, and a substantial reduction in lung metastases (Fig. 6). These in vivo effects on metastastic reduction are very similar to the in vivo anti-metastatic effects of non-encapsulated cytochalasin B (demonstrated in this study [4, 16]). The reduced immunosuppression by liposomal cytochalasin B with retention of anti-metastatic properties suggests that the liposomal formulation may be superior to non-encapsulated cytochalasin B for treatment of metastasizing or metastatic neoplasms. It was also shown that two 90 mg/kg i.p. injections, separated 5 days apart, can mitigate further tumor growth of i.d. nodules and can produce tumor shrinkage (Fig. 7).

This study demonstrates that cytochalasin B produces both localized, separate compartment, and systemic antitumor activity against established tumor nodules. The activity is evidenced by significant alterations to tumor growth rate, invasion, metastases, host survival time, and in some cases, complete tumor regression. Direct gross toxicity is unlikely to be responsible for the effects observed, although toxic effects on sensitive clonogenic subpopulations of tumor cells are not excluded by these data. The localized and systemic perturbations of solid tumors, and the long-delayed influence observed with i.v. drug treatment suggest that direct toxicity is not the sole, or even the most important mechanism by which cytochalasin B exerts in vivo activity. Therefore, it appears plausible that the effects of cytochalasin B in vivo depend on features of tumor tissue physiology, as well as the host response to solid tumors. In addition, liposomal cytochalasin B appears to be an attractive formulation of the agent, exhibiting decreased host toxicity and increased efficacy with favorable effects on host immune response. The liposomal formulation of cytochalasin B and of other cytochalasin congeners may increase the opportunity to develop microfilament-directed agents that are of value in the clinical management of neoplasms.
